# Cannabinoid CB2 receptors are upregulated *via* bivalent histone modifications and control primary afferent input to the spinal cord in neuropathic pain

**DOI:** 10.1016/j.jbc.2022.101999

**Published:** 2022-04-29

**Authors:** Krishna Ghosh, Guang-Fen Zhang, Hong Chen, Shao-Rui Chen, Hui-Lin Pan

**Affiliations:** Center for Neuroscience and Pain Research, Department of Anesthesiology and Perioperative Medicine, The University of Texas MD Anderson Cancer Center, Houston, Texas, USA

**Keywords:** cannabinoid receptor, *Cnr2*, epigenetics, histone acetylation, histone methylation, synaptic plasticity, 5-mC, 5-methylcytosine, aCSF, artificial cerebrospinal fluid, BSP, bisulfite sequencing PCR, CB2, Type-2 cannabinoid receptors, ChIP-qPCR, chromatin immunoprecipitation–quantitative PCR, DRG, dorsal root ganglion, EPSCs, excitatory postsynaptic currents, GLP, G9a-like protein, HRP, horseradish peroxidase, MeDIP, methylated DNA immunoprecipitation, PPR, paired-pulse ratio, qPCR, quantitative PCR, SNL, spinal nerve ligation, TBST, Tris-buffered saline and Tween 20, TSS, transcription start site

## Abstract

Type-2 cannabinoid receptors (CB2, encoded by the *Cnr2* gene) are mainly expressed in immune cells, and CB2 agonists normally have no analgesic effect. However, nerve injury upregulates CB2 in the dorsal root ganglion (DRG), following which CB2 stimulation reduces neuropathic pain. It is unclear how nerve injury increases CB2 expression or how CB2 activity is transformed in neuropathic pain. In this study, immunoblotting showed that spinal nerve ligation (SNL) induced a delayed and sustained increase in CB2 expression in the DRG and dorsal spinal cord synaptosomes. RNAscope *in situ* hybridization also showed that SNL substantially increased CB2 mRNA levels, mostly in medium and large DRG neurons. Furthermore, we found that the specific CB2 agonist JWH-133 significantly inhibits the amplitude of dorsal root–evoked glutamatergic excitatory postsynaptic currents in spinal dorsal horn neurons in SNL rats, but not in sham control rats; intrathecal injection of JWH-133 reversed pain hypersensitivity in SNL rats, but had no effect in sham control rats. In addition, chromatin immunoprecipitation–qPCR analysis showed that SNL increased enrichment of two activating histone marks (H3K4me3 and H3K9ac) and diminished occupancy of two repressive histone marks (H3K9me2 and H3K27me3) at the *Cnr2* promoter in the DRG. In contrast, SNL had no effect on DNA methylation levels around the *Cnr2* promoter. Our findings suggest that peripheral nerve injury promotes CB2 expression in primary sensory neurons *via* epigenetic bivalent histone modifications and that CB2 activation reduces neuropathic pain by attenuating nociceptive transmission from primary afferent nerves to the spinal cord.

Neuropathic pain is a chronic debilitating condition, and current treatments are unsatisfactory. Endogenous endocannabinoids, including N-arachidonoylethanolamine and 2-arachidonoylglycerol, produce biological effects mainly through activation of two cannabinoid receptors, type-1 cannabinoid receptors (CB1, encoded by the *Cnr1* gene) and type-2 cannabinoid receptors (CB2, encoded by *Cnr2*) (1). CB1 are widely distributed in the peripheral and central nervous system (2, 3). Although CB1 activation produces analgesia in several painful conditions (4, 5), CB1 agonists are not suitable analgesics for clinical use because of their severe adverse effects, such as addiction. Unlike CB1, CB2 are predominantly present in immune cells, including microglia and mast cells but are poorly expressed in the nervous system (6, 7). Interestingly, peripheral nerve injury increases CB2 expression in the dorsal root ganglion (DRG) (5, 8). Furthermore, CB2 agonists have no analgesic effect under normal conditions but produce potent analgesic effects in animal models of neuropathic pain (9, 10, 11, 12). However, it is unclear how the CB2 action is transformed in neuropathic pain and how nerve injury upregulates CB2 in the DRG.

Nerve injury causes distinct upregulation and downregulation of a large number of gene targets, which participate in long-lasting hyperexcitability of primary afferent nerves and DRG neurons and persistent nociceptive input to the spinal cord (13, 14). Epigenetic mechanisms, such as histone posttranslational modifications (*e.g.*, methylation and acetylation) and DNA methylation, play a critical role in the development of hyperexcitability of DRG neurons and chronic pain after nerve injury. For example, nerve injury increases the expression and activity of G9a, a histone lysine methyltransferase, which represses transcription of antinociceptive genes, including CB1, μ-opioid receptors, and potassium channels, by increasing enrichment of H3 lysine 9 dimethylation (H3K9me2, a repressive histone mark) at these gene promoters in DRG neurons (5, 14, 15). Consequently, mice with G9a ablated from DRG neurons do not develop chronic pain after nerve injury (14, 15). Furthermore, nerve injury reduces enrichment of H3 lysine 9 acetylation (H3K9ac, an activating histone mark) at the gene promoters of potassium channels and μ-opioid receptors but increases H3K9ac abundance at the pannexin-1 gene promoter in the DRG in neuropathic pain (14, 15, 16). In addition, nerve injury causes dynamic, genome-wide reprogramming of DNA methylation in the DRG (17). However, it is uncertain whether histone modifications and/or DNA methylation play a role in the transcriptional regulation of CB2 expression in the DRG in chronic neuropathic pain.

Therefore, in the present study, we determined CB2 expression in DRG neurons and its functional significance in regulating nociceptive transmission at the spinal cord level in neuropathic pain. We also determined epigenetic changes associated with active transcription of *Cnr2* in the injured DRG. Consistent with previous reports (5, 8), our study demonstrates that nerve injury induces a long-lasting increase in CB2 expression in DRG neurons, which inhibits glutamatergic input from primary afferents to spinal dorsal horn neurons in neuropathic pain. Furthermore, nerve injury–induced CB2 upregulation is associated with divergent histone modifications, but not DNA methylation, at the *Cnr2* promoter in the DRG. This new knowledge advances our mechanistic understanding of CB2 expression in primary sensory neurons and CB2-mediated analgesic actions in neuropathic pain.

## Results

### Nerve injury increases CB2 expression in DRG neurons

**Figure 1:**
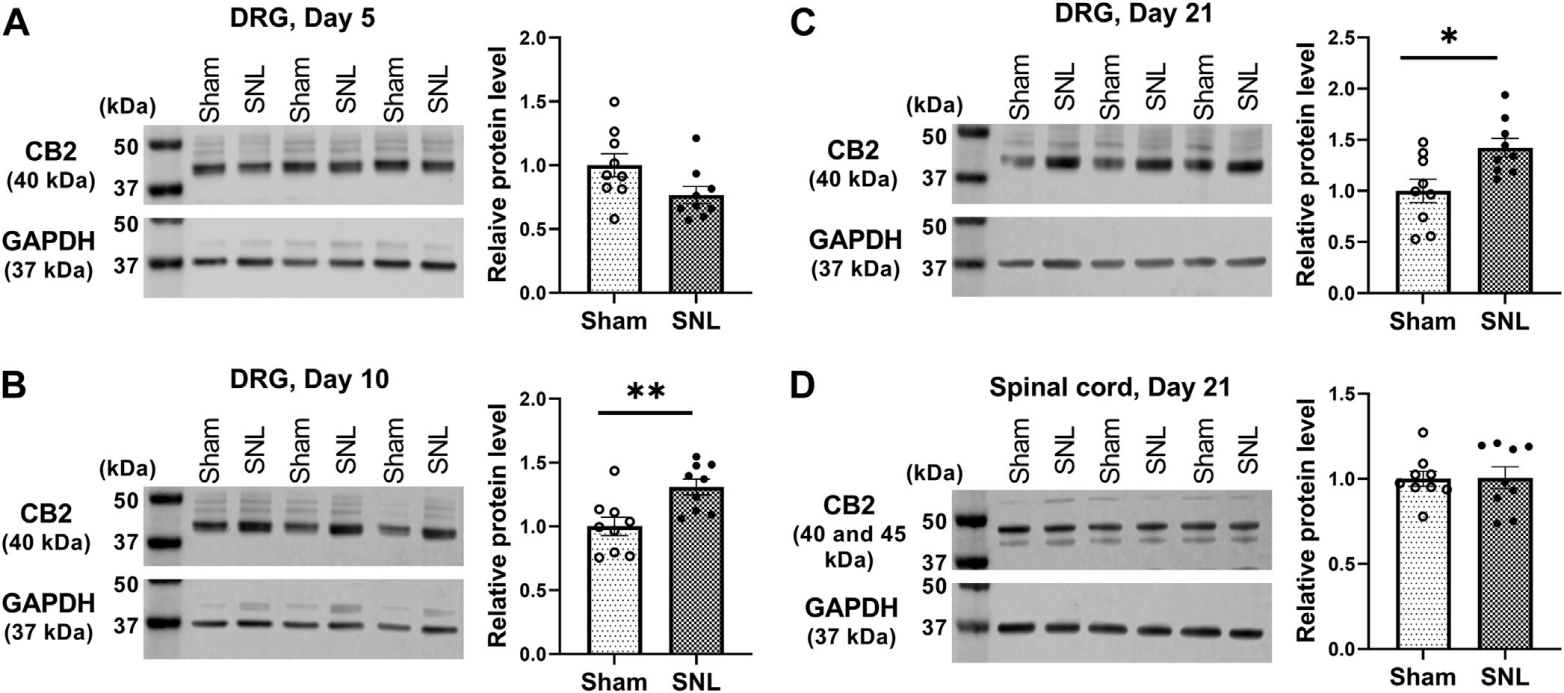
**Nerve injury causes a delayed and sustained increase in CB2 expression in the DRG.***A*–*C*, representative gel images and quantification of CB2 protein level in the rat DRG 5 (*A*), 10 (*B*), and 21 (*C*) days after sham or SNL surgery. *D*, representative gel images and quantification of CB2 protein level in the rat dorsal spinal cord 21 days after sham or SNL surgery. Data are shown as means ± SEM (n = 9 rats per group). GAPDH was used as endogenous loading control, and the mean value in the sham group was normalized to 1. ∗*p* < 0.05, ∗∗*p* < 0.01, compared with the sham group (Two-tailed Student’s *t* test). CB2, Type-2 cannabinoid receptors; DRG, dorsal root ganglion; SNL, spinal nerve ligation.

We first used immunoblotting to determine the time course of changes in the expression levels of CB2 in the DRG after nerve injury. Immunoblotting assays showed that compared with that in sham rats, the protein level of CB2 in the DRG was significantly increased in spinal nerve ligation (SNL) rats at day 10 (t(16) = 3.240, *p* = 0.005) and day 21 (t(16) = 2.893, *p* = 0.011), but not day 5, after surgery (n = 9 rats per group; Fig. 1, *A*–*C*). However, SNL had no effect on the protein level of CB2 in the dorsal spinal cord 21 days after surgery (n = 9 rats per group; t(16) = 0.080, *p* = 0.937; Fig. 1*D*).

**Figure 2:**
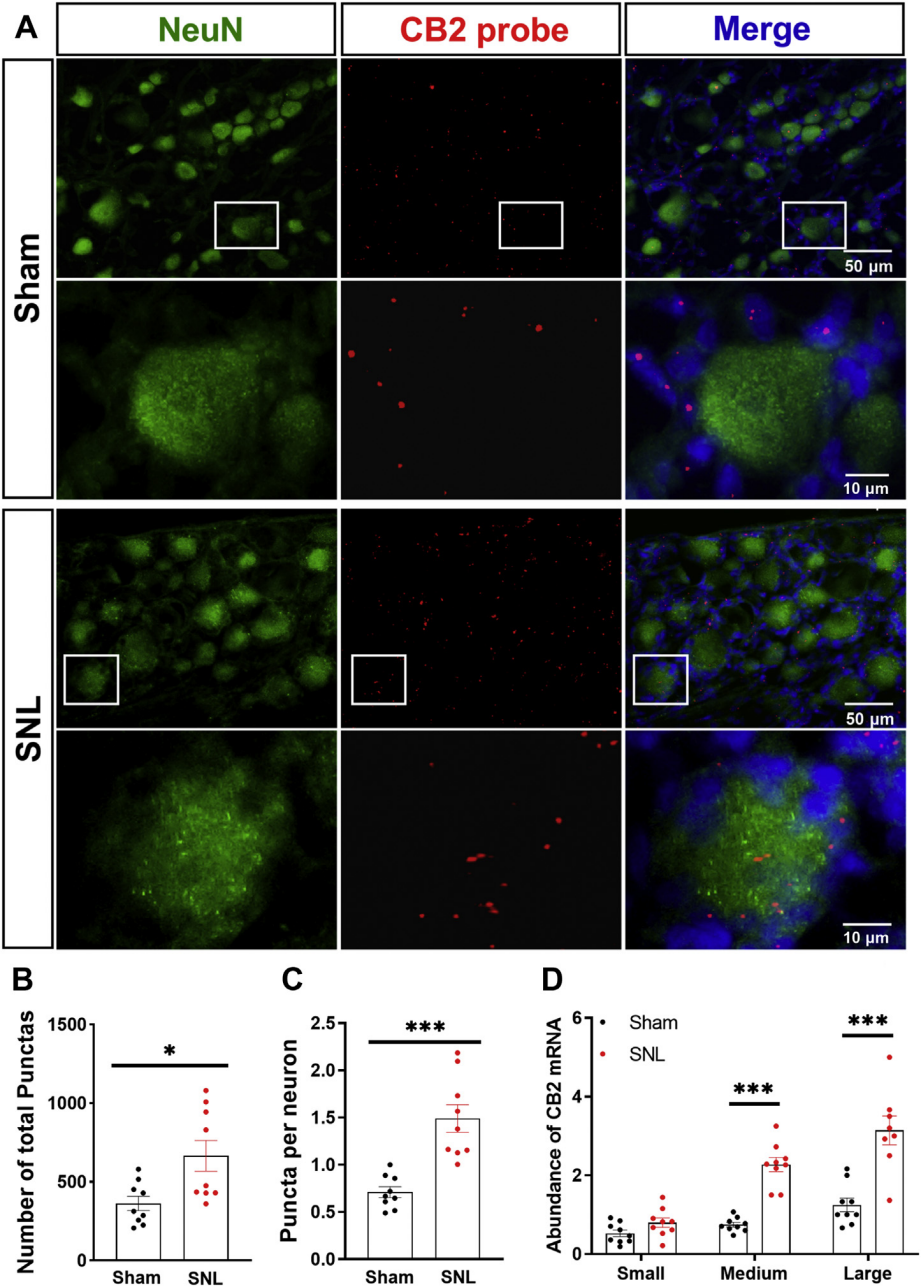
**Nerve injury increases CB2 expression in DRG neurons.***A*, representative low- and high-magnification images of RNAscope *in situ* hybridization show colabeling of NeuN (*green*) and CB2 mRNA (*red*) in DRG tissue sections from sham and SNL rats 10 days after surgery. *B*–*D*, quantification of CB2 mRNA punctate signals in the DRG tissue section (*B*), the abundance of CB2 mRNA signals in DRG neurons (*C*), and amount of CB2 mRNA signals in small-, medium-, and large-diameter DRG neurons (*D*) in sham and SNL rats. Data are shown as means ± SEM (n = 9 images per group). ∗*p* < 0.05, ∗∗∗*p* < 0.001 compared with the sham group (Two-tailed Student’s *t* test). CB2, Type-2 cannabinoid receptors; DRG, dorsal root ganglion; SNL, spinal nerve ligation.

Although CB2 is predominantly present in immune cells under normal conditions (7), CB2 may be expressed in DRG neurons after nerve injury (8). Because the CB2 antibody used for immunoblotting produced nonspecific labeling in our preliminary studies, we used RNAscope to determine the expression of CB2 mRNA in DRG neurons 10 days after nerve injury. The CB2 punctate signal was detected in many NeuN-labeled neurons and some NeuN-negative cells (probably satellite glial cells). The total number of punctate signals of CB2 mRNA in the DRG section was significantly greater in SNL rats than in sham rats (t(16) = 2.81, *p* = 0.013, n = 9 per group; Fig. 2, *A* and *B*). Also, the abundance of CB2 mRNA punctate signals in NeuN-labeled DRG neurons was significantly increased in SNL rats compared with that in sham rats (t(16) = 5.62, *p* < 0.001; n = 9 per group; Fig. 2, *A* and *C*). Furthermore, the abundance of CB2 mRNA punctate signals was significantly greater in medium-diameter and large-diameter, but not in small-diameter, DRG neurons in SNL rats than in sham rats (Fig. 2*D*). These results indicate that peripheral nerve injury induces sustained upregulation of CB2 in primary sensory neurons.

### CB2 activation reverses SNL-induced glutamatergic input from primary afferents to spinal cord

**Figure 3:**
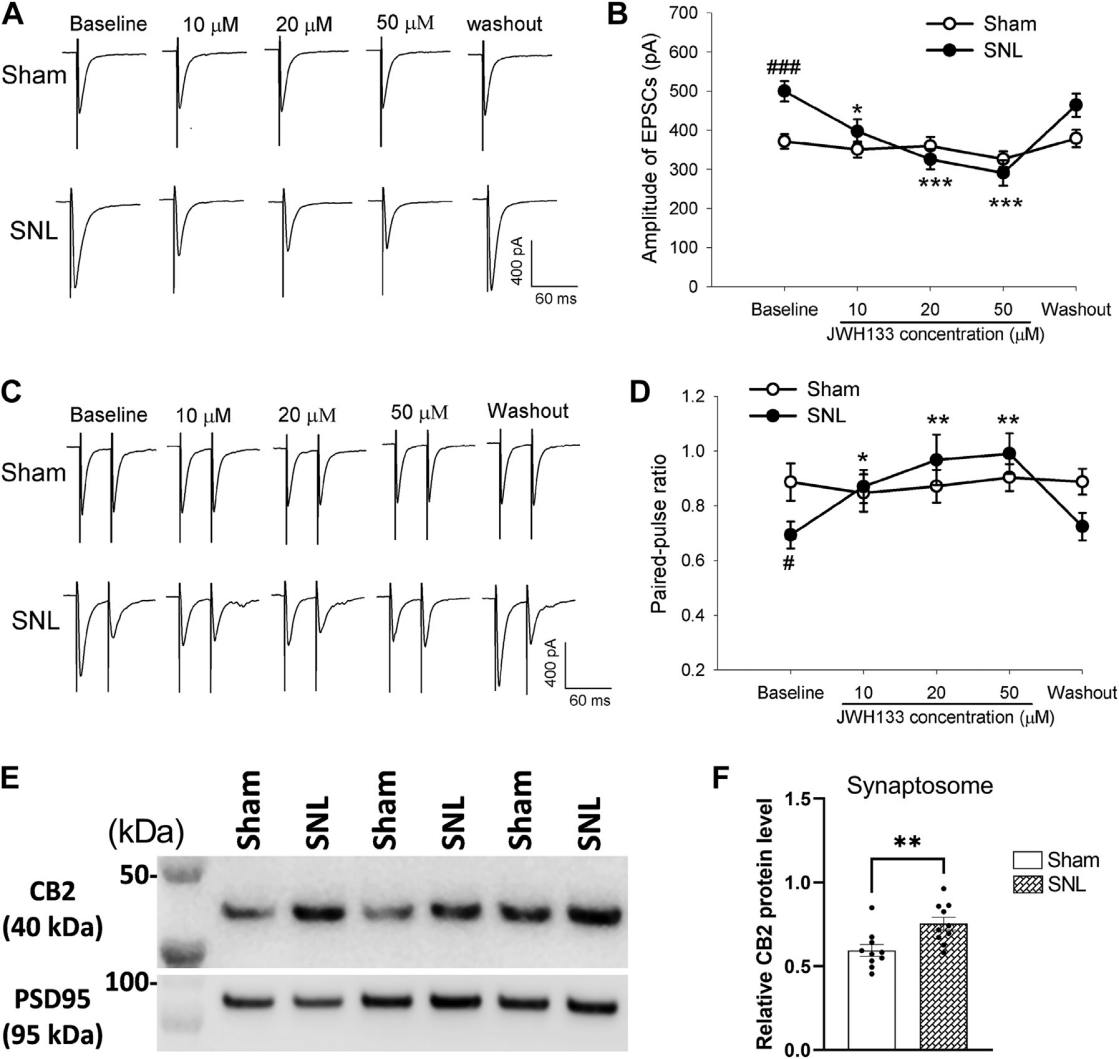
**CB2 stimulation preferentially inhibits glutamatergic input from primary afferents to spinal dorsal horn neurons increased by nerve injury.***A* and *B*, representative recording traces (*A*) and mean data (*B*) show the effect of bath application of 10, 20, and 50 μM JWH-133 on the amplitude of EPSCs of dorsal horn neurons evoked monosynaptically from dorsal root stimulation in four sham (n = 12 neurons) or five SNL (n = 13 neurons) rats 2 to 3 weeks after surgery. *C* and *D*, original recording traces (*C*) and mean data (*D*) show the effect of bath application of 10 to 50 μM JWH-133 on the paired-pulse ratio of dorsal horn neurons in four sham (n = 11 neurons) or five SNL (n = 13 neurons) rats 2 to 3 weeks after surgery. Data are shown as means ± SEM. In (B), two-way ANOVA showed that there was a significant main effect for drug treatment (F(4,48) = 19.04, *p* < 0.001) and a significant interaction between SNL and sham conditions (F(12,48) = 6.27, *p* < 0.001). In (D), two-way ANOVA showed that there was a significant main effect for drug treatment (F(4,48) = 6.65, *p* < 0.001) and a significant interaction between SNL and sham conditions (F(12,48) = 5.56, *p* < 0.001). ^#^*p* < 0.05, ^###^*p* < 0.001, compared with the baseline in sham rats. ∗*p* < 0.05, ∗∗*p* < 0.01, ∗∗∗*p* < 0.001, compared with the baseline in SNL rats (two-way ANOVA followed by the Dunnett’s *post hoc* test). *E* and *F*, representative blotting images (*E*) and quantification (*F*) of the CB2 protein level in dorsal spinal cord synaptosomes in SNL and sham control rats 21 days after surgery (n = 10 rats per group). Data are shown as mean ± SEM. PSD95, a synaptic protein, was used as a loading control. ∗∗*p* < 0.01 (Two-tailed Student’s *t* test). CB2, Type-2 cannabinoid receptors; EPSCs, excitatory postsynaptic currents; SNL, spinal nerve ligation.

CB2 activation at the spinal cord level can reduce neuropathic pain (8, 18). We next performed whole-cell voltage-clamp recordings in spinal cord slices to determine the functional significance of CB2 at primary afferent central terminals in the control of glutamatergic input to the spinal cord in SNL and sham rats 2 to 3 weeks after surgery. We recorded excitatory postsynaptic currents (EPSCs) of lamina II neurons monosynaptically evoked by electrical stimulation of the dorsal root. Similar to what we reported previously (19, 20), the baseline amplitude of evoked EPSCs was significantly higher in SNL than in sham rats (t(23) = 3.931, *p* < 0.001; Fig. 3, *A* and *B*). In sham rats, bath application of JWH-133, a specific CB2 agonist (21, 22), at concentrations of 10 to 50 μM had no effect on the amplitude of evoked EPSCs of spinal lamina II neurons (n = 12 neurons; Fig. 3, *A* and *B*). In contrast, in SNL rats, bath application of 10 to 50 μM of JWH-133 significantly reduced the amplitude of evoked monosynaptic EPSCs of lamina II neurons in a concentration-dependent manner (n = 13 neurons; Fig. 3, *A* and *B*).

The baseline paired-pulse ratio (PPR) of evoked EPSCs, a measure of presynaptic glutamate release (23, 24), was significantly lower in SNL than in sham rats (t(22) = 2.454, *p* = 0.024; Fig. 3, *C* and *D*). In SNL rats, bath application of 10 to 50 μM JWH-133 produced a greater reduction in the amplitude in the first EPSC than the second EPSC, resulting in a significant increase in the PPR of evoked EPSCs in lamina II neurons (n = 13 neurons; Fig. 3, *C* and *D*). However, JWH-133 at 10 to 50 μM had no significant effect on the PPR of evoked EPSCs in spinal lamina II neurons in sham rats (n = 11 neurons; Fig. 3, *C* and *D*).

In addition, we obtained synaptosomes from the dorsal spinal cord tissues to determine whether nerve injury increases CB2 synaptic trafficking. Immunoblotting showed that CB2 proteins in spinal cord synaptosomes was significantly greater in SNL rats than in sham control rats 3 weeks after surgery (t(18) = 3.18, *p* = 0.006, n = 10 rats per group; Fig. 3, *E* and *F*). Collectively, these data suggest that activation of presynaptic CB2 at primary afferent central terminals augmented by nerve injury inhibits glutamatergic input to spinal dorsal horn neurons.

### Activating CB2 at the spinal cord level reduces SNL-induced pain hypersensitivity

**Figure 4:**
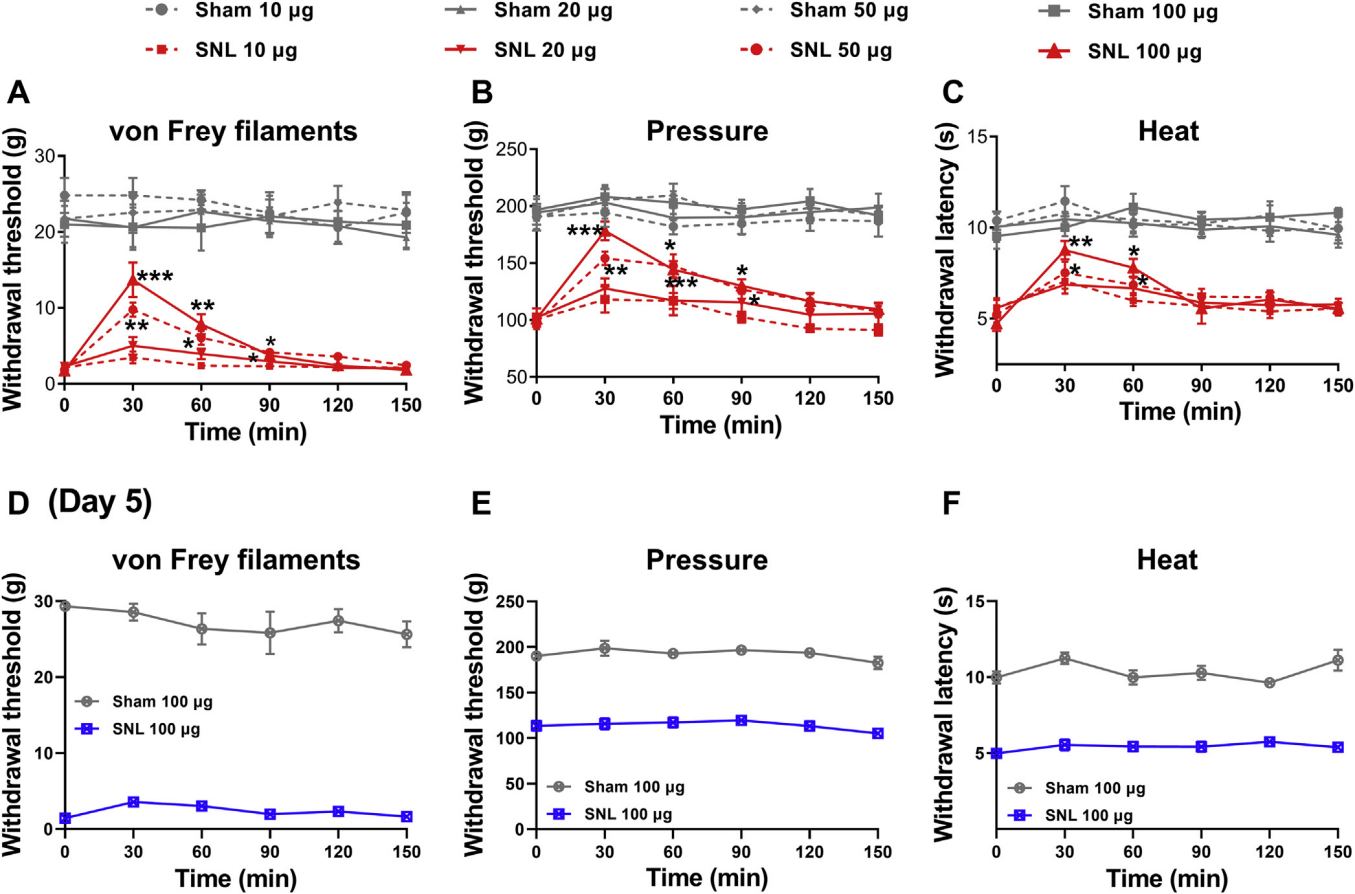
**CB2 activation at the spinal cord level attenuates pain hypersensitivity induced by nerve injury.***A*–*C*, time course of the effect of intrathecal injection of 10 to 100 μg JWH-133 on the paw withdrawal thresholds tested using von Frey filaments (A; 50 μg: *p* < 0.001, F(1.88,13.14) = 26.83; 100 μg: *p* < 0.001, F(1.26,8.84) = 22.82), a pressure stimulus (B; 50 μg: *p* < 0.001, F(2.52, 17.61) = 19.68; 100 μg: *p* < 0.001, F(2.65,18.54) = 26.30), and a heat stimulus (C; 50 μg: *p* = 0.0017, F(2.90,20.30) = 7.40; 100 μg: *p* < 0.001, F(2.97,20.81) = 10.95) in rats 2 to 3 weeks after sham or SNL surgery (n = 8 animals per group). Data are expressed as means ± SEM. ∗*p* < 0.05, ∗∗*p* < 0.01, ∗∗∗*p* < 0.001, compared with the respective baseline before drug injection (time 0; repeated measures ANOVA followed by the Dunnett’s *post hoc* test). *D*–*F*, lack of an effect of intrathecal injection of 100 μg JWH-133 on paw withdrawal thresholds tested using von Frey filaments (*D*), a pressure stimulus (*E*), and a heat stimulus (*F*) in rats 5 days after sham or SNL surgery (n = 7 rats per group). CB2, Type-2 cannabinoid receptors; SNL, spinal nerve ligation.

We then used the CB2 agonist JWH-133 to determine the role of CB2 in the spinal cord level in the control of nerve injury–induced neuropathic pain. Agents injected *via* intrathecal catheter can access directly to both the DRG and spinal cord (25, 26). In sham and SNL rats 2 to 3 weeks after surgery, we tested the effect of intrathecal injection of 10, 20, 50, and 100 μg of JWH-133 on the withdrawal thresholds of hindpaw in response to tactile, mechanical nociceptive, and heat stimuli. In SNL rats, intrathecal administration of 50 and 100 μg, but not 10 or 20 μg, of JWH-133 significantly attenuated tactile allodynia, mechanical hyperalgesia, and heat hyperalgesia (n = 8 rats per group; Fig. 4, *A*–*C*). However, in sham control rats, intrathecal injection of 10 to 100 μg of JWH-133 had no significant effect on tactile, pressure, or heat withdrawal thresholds (n = 8 rats per group; Fig. 4, *A*–*C*). Furthermore, intrathecal injection of 100 μg of JWH-133 had no significant effect on pain hypersensitivity 5 days after SNL surgery, consistently with our finding showing no increased CB2 expression at day 5 after nerve injury (Fig. 4, *D*–*F*). These data suggest that CB2 activation at the spinal cord level attenuates peripheral nerve injury–induced neuropathic pain at the time only when CB2 expression is increased in DRG neurons and their central terminals.

### Nerve injury alters histone modifications at the Cnr2 promoter in the DRG

**Figure 5:**
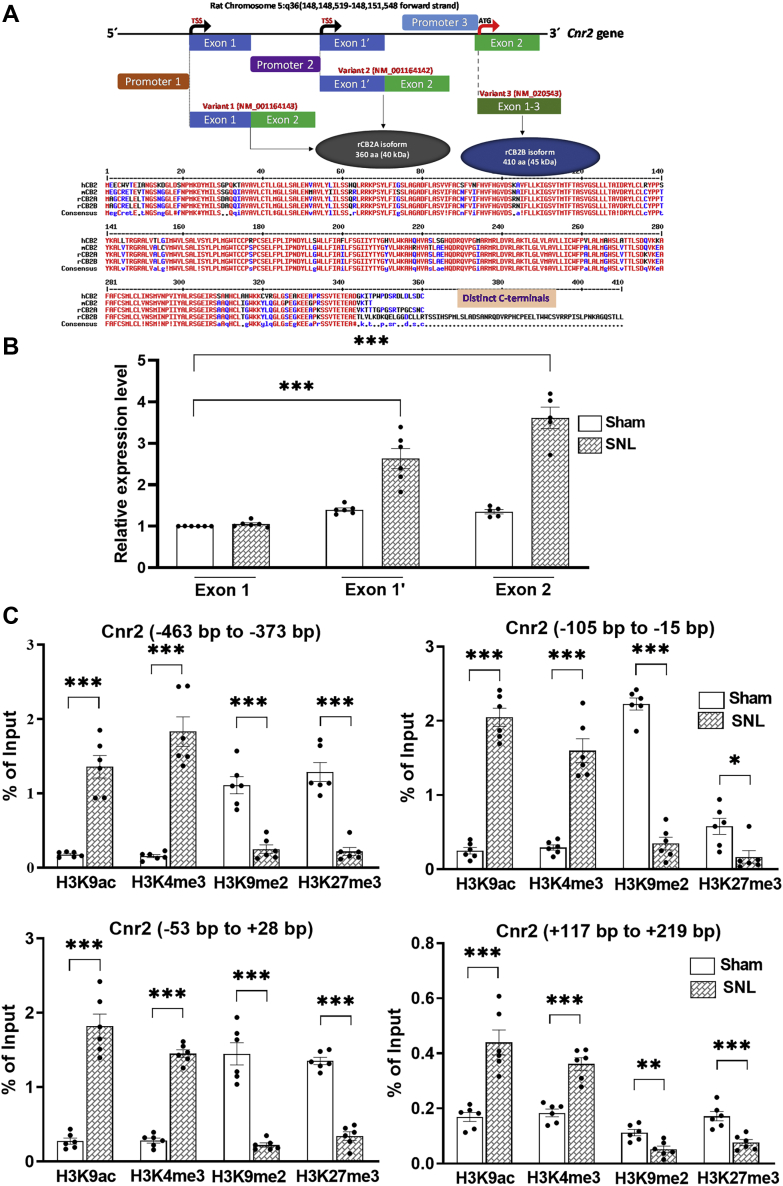
**Nerve injury alters activating and repressive histone modifications at the *Cnr2* promoter in the DRG.***A*, *top*, schematic representation of the rat *Cnr2* gene exon and intron composition of different transcript variants. The coding sequence (starting with ATG) of the same 360 amino acid (aa) long CB2 protein (rCB2A) is localized solely within the common Exon 2 of both transcript variant 1 and variant 2. Transcript variant 3 (Exon 1–3), although 100% identical to Exon 2, encodes a longer (410 aa) CB2 protein (rCB2B). *Bottom*, alignment of rat CB2 protein isoforms with corresponding human (hCB2) and mouse (mCB2) isoforms shows the presence of a distinct C-terminal sequence in the rCB2B isoform, whereas the rCB2A isoform shares a high degree of similarity with hCB2 and mCB2. *B*, real-time qPCR analysis of Cnr2 mRNA levels using specific primers for each indicated Exon in rat DRG tissues 21 days after sham or SNL surgery. Data are expressed as mean ± SEM (n = 6 independent experiments; *p* < 0.0001, F(5,30) = 53.36). ∗∗∗*p* < 0.001 compared with the sham group of Exon 1 (one-way ANOVA followed by Dunnett’s *post hoc* test). *C*, ChIP-qPCR quantification shows the enrichment of histone marks at -463 bp to -373 bp, -115 bp to -15 bp, -53 bp to +28 bp, and +117 bp to +219 bp regions flanking the transcription start site of the rat *Cnr2* gene on chromosome 5. DRG tissues were obtained from rats 21 days after sham or SNL surgery and subjected to ChIP-qPCR to quantify the immunoprecipitated DNA using the antibodies against H3K9ac, H3K4me3, H3K9me2, H3K27me3, and pan-histone H3. The percentage of their enrichment at each indicated region on the *Cnr2* promoter was normalized to the input sample and corrected with that of pan-histone H3 values. Rabbit IgG was used as a negative control in immunoprecipitations. The rat negative control primers spanning a gene desert on rat chromosome 3 was used to confirm the specificity of ChIP-qPCR. Data are expressed as means ± SEM (n = 6 independent experiments per group; DRG tissues from three rats were pooled as one sample). ∗*p* < 0.05, ∗∗*p* < 0.01, ∗∗∗*p* < 0.001, compared with the sham group (two-tailed Student’s *t* test). CB2, Type-2 cannabinoid receptors; ChIP-qPCR, chromatin immunoprecipitation–quantitative PCR; DRG, dorsal root ganglion; SNL, spinal nerve ligation.

Nerve injury–induced gene expression in the DRG is often associated with altered histone modifications at the gene promoter regions (5, 14, 15, 16). We next determined whether nerve injury–induced CB2 upregulation in the DRG involves histone modifications. We first analyzed rat *Cnr2* transcript variants (Fig. 5*A*) reported in the NCBI Gene database (Gene ID: 57302). Because the distinct transcription start sites (TSS) usually indicate the presence of different promoters, we hypothesized that at least two independent promoters of *Cnr2* exist in the rat. The transcript variant 1 and variant 2 have distinct first exons encoded from two distal DNA sequences on chromosome 5. However, both variants have 100% identical second exon. Although both variant 1 and variant 2 transcripts encode the same 360-amino acid long CB2 protein (referred to as rCB2A isoform), the transcript variant 3 has a long CB2 isoform with a distinct C terminus (rCB2B isoform). The *Cnr2* transcription in humans and mice is controlled *via* at least two promoters, leading to a tissue-specific expression of two different *Cnr2* isoforms (CB2A and CB2B) (27). Sequence alignment of rCB2 isoforms with human and mouse CB2 proteins revealed a very close similarity between rCB2A with hCB2 and mCB2 (Fig. 5*A*). Our immunoblotting results confirmed a DRG-specific expression of a short rCB2A isoform (40 kDa); but in the spinal cord, both CB2 isoforms (40 kDa and 45 kDa) are present (Fig. 1). Furthermore, real-time PCR analysis using exon-specific primers showed that the variant 2 (Exon 1′) is predominantly expressed in the DRG and that the transcript of its promoter (promoter 2) was significantly increased by nerve injury (Fig. 5*B*). Thus, we selected this *Cnr2* promoter for our chromatin immunoprecipitation–quantitative PCR (ChIP-qPCR) assays.

The presence of functionally opposite histone marks at gene promoters is defined as ‘bivalency’, which mainly mark development-related genes in embryonic stem cells (28). Histone H3 lysine 9 dimethylation (H3K9me2) and H3 lysine 27 trimethylation (H3K27me3) are associated with genes in a repressed state, whereas H3 lysine 4 trimethylation (H3K4me3) and H3 lysine 9 acetylation (H3K9ac) are generally linked to genes with active expression (14, 29). We used ChIP-qPCR to determine the enrichment of two activating histone marks, H3K9ac and H3K4me3, and two silencing histone marks, H3K9me2 and H3K27me3, at the *Cnr2* promoter in L5 and L6 DRGs 21 days after SNL or sham surgery. Four different regions spanning from 463 bp upstream to 219 bp downstream surrounding the TSS at the rat *Cnr2* promoter were analyzed for enrichment of these histone marks. The enrichment of H3K9ac in all four regions at the *Cnr2* promoter in the L5 and L6 DRGs was significantly greater in SNL rats than in sham rats (n = 6, Fig. 5*C*). Among the four regions analyzed, H3K9ac was most highly enriched at the -105 bp to -15 bp region (t(10) = 13.75, n = 6 samples per group, *p* < 0.001) and at the -53 bp to +28 bp region (t(10) = 9.316, n = 6 samples per group, *p* < 0.001) of the *Cnr2* promoter in SNL rats compared to sham rats. Also, the enrichment of H3K4me3 across all four regions at the *Cnr2* promoter was significantly increased in in the DRG of SNL rats compared with that in sham control rats (Fig. 5*C*). The H3K4me3 was highly enriched in the -463 bp to -373 bp region (t(10) = 8.387, n = 6 samples per group, *p* < 0.001) and in the -105 bp to -15 bp region (t(10) = 7.960, n = 6 samples per group, *p* < 0.001).

In contrast, the occupancy of the repressive histone mark H3K9me2 at the *Cnr2* promoter in the DRG was significantly reduced by nerve injury in the -463 bp to -373 bp region (t(10) = 6.655, n = 6 samples per group, *p* < 0.001), in the -105 bp to -15 bp region (t(10) = 15.94, n = 6 samples per group, *p* < 0.001), and in the -53 bp to +28 bp region (t(10) = 8.077, n = 6 samples per group, *p* < 0.001) (Fig. 5*C*). Furthermore, nerve injury significantly reduced the enrichment of H3K27me3 across all four regions at the *Cnr2* promoter (n = 6, Fig. 5*C*). These findings suggest that nerve injury–induced CB2 upregulation in the DRG likely results from a coordinated increase in activating histone marks and reduction in repressive histone marks at the *Cnr2* promoter.

### Nerve injury does not alter DNA methylation status at the Cnr2 promoter in the DRG

**Figure 6:**
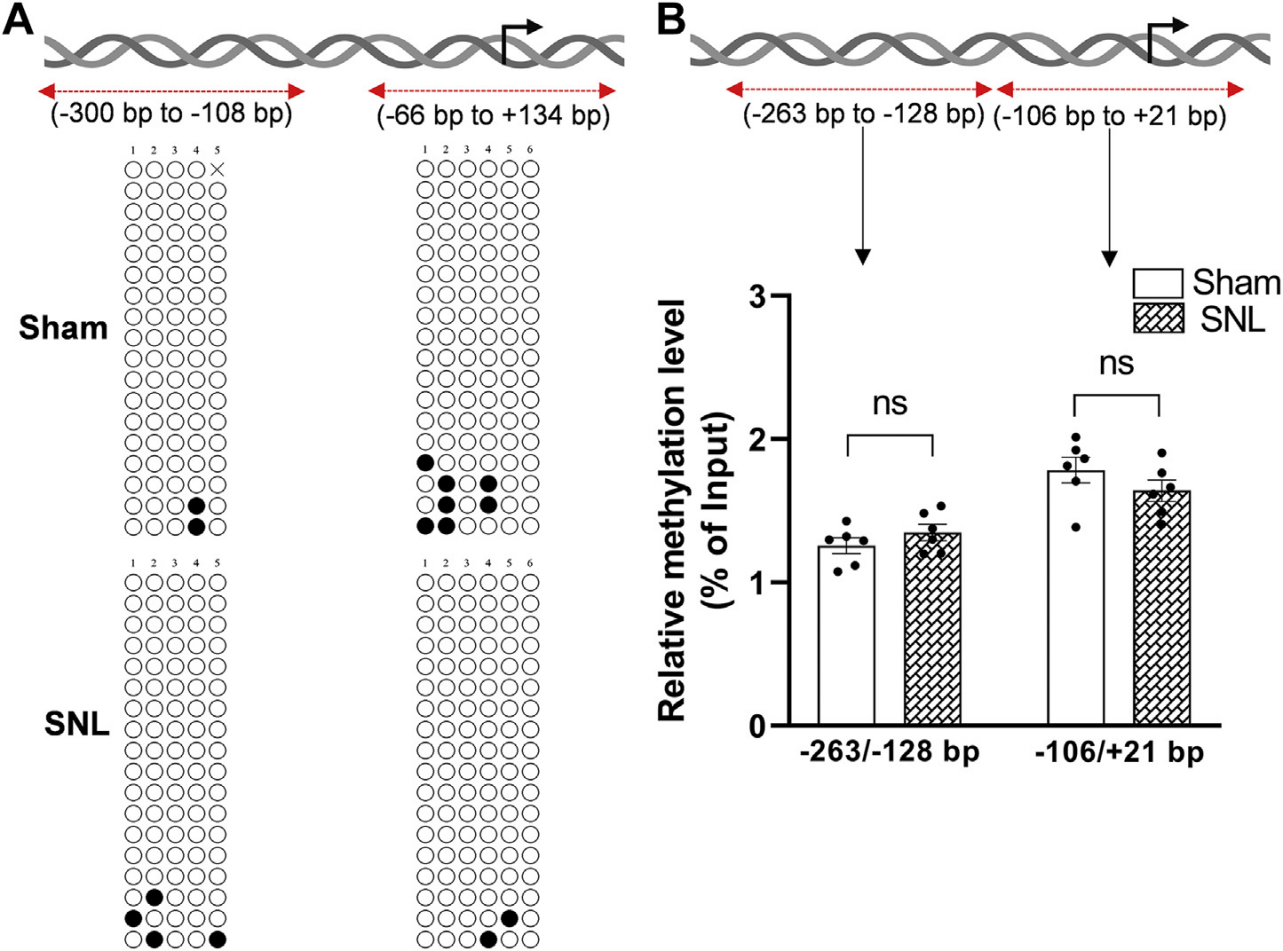
**Nerve injury does not alter the DNA methylation level at the rat *Cnr2* promoter in the DRG.***A*, bisulfite sequencing PCR (BSP) analysis of the *Cnr2* promoter using DNA obtained from the L5 and L6 DRG of sham and SNL rats 21 days after surgery. The rat *Cnr2* promoter, TSS (*black arrowhead*), and the PCR amplified regions (-300 bp to -108 bp and -66 bp to +134 bp) are schematically represented at the *top*. *Open circles* at the bottom scheme represent unmethylated CpG sites, and *filled circles* represent methylated CpG dinucleotides within the cloned regions at the *Cnr2* promoter. Each row represents the CpG methylation in a clone (n = 3 independent experiments with a total of 18 clones sequenced in each group). *B*, MeDIP-qPCR analysis of the enrichment of 5-mC at the respective *Cnr2* promoter region in the DRG of sham and SNL rats 21 days after surgery (the percentage of 5-mC enrichment relative to the input in each group; n = 6 independent experiments per group; DRG tissues from three rats were pooled as one sample). Data are expressed as means ± SEM (ns, not significantly different; Mann-Whitney U test). DRG, dorsal root ganglion; MeDIP-qPCR, methylated DNA immunoprecipitation–quantitative PCR; SNL, spinal nerve ligation; TSS, transcription start site.

Because nerve injury also causes DNA methylation reprogramming in the DRG (17) and because DNA methylation controls gene expression (30), we conducted additional experiments to determine whether nerve injury alters DNA methylation status of *Cnr2* in the DRG. We used bisulfite sequencing PCR (BSP) to assess the methylation status of CpG sites at the rat *Cnr2* promoter from -300 bp to -108 bp and -66 bp to +134 bp in L5 and L6 DRGs 21 days after SNL or sham surgery. The proximal promoter of *Cnr2* is devoid of any dense CpG islands in the MethPrimer prediction. Our BSP assay showed that SNL did not alter the methylation status of CpG sites at the *Cnr2* promoter in the DRG (Fig. 6*A*). The CpG sites within the -300 bp to -108 bp region and -66 bp to +143 bp region, spanning the TSS of *Cnr2*, in the DRG remained largely unmethylated in SNL and sham groups (Fig. 6*A*).

Because bisulfite conversion does not distinguish between 5-methylcytosine (5-mC) and 5-hydroxymethylcytosine, we also conducted methylated DNA immunoprecipitation (MeDIP) using a highly specific 5-mC antibody (31) followed by quantitative PCR (qPCR) analysis. There were no significant differences in the enrichment of 5-mC across the -263 bp to -128 bp region (t(10) = 1.15, *p* = 0.275) or the -106 bp to +21 bp region (t(10) =1.23, *p* = 0.245) at the rat *Cnr2* promoter in the DRG between sham and SNL groups (n = 6, Fig. 6*B*). These results suggest that despite the presence of discrete CpG sites around the TSS of *Cnr2*, nerve injury–induced CB2 upregulation in the DRG is unlikely caused by the altered DNA methylation level at the *Cnr2* promoter.

## Discussion

In the present study, we found that nerve injury caused a delayed and sustained increase in CB2 expression in the DRG. CB2 is poorly expressed in the DRG under normal conditions but is upregulated after nerve injury (5, 8, 32). To study nerve injury–induced CB2 expression specifically in primary sensory neurons, we used RNAscope *in situ* hybridization to determine the CB2 mRNA levels in DRG neurons. We showed that nerve injury markedly increased the CB2 mRNA level in medium- and large-sized DRG neurons. Furthermore, we found that intrathecal injection of the CB2 agonist JWH-133 effectively reversed the reduction in the withdrawal thresholds measured with tactile, noxious pressure, and heat stimuli in SNL rats when CB2 is upregulated in the DRG but had no such effects in sham control rats and SNL rats 5 days after nerve ligation. These data suggest that CB2 activation at the spinal cord level does not affect normal nociception but preferentially reduces neuropathic pain caused by nerve injury. JWH-133 is a selective CB2 agonist with a Ki of 3.4 nM and has a 200-fold selectivity for CB2 over CB1 (21, 22). The inhibitory effect of JWH-133 is abolished by the CB2 antagonist SR144528 and is absent in CB2 KO mice (33, 34). Our findings are consistent with previous studies showing that selective CB2 agonists reduce neuropathic pain (9, 11, 12). Because intrathecally administered drugs can access directly to DRG neurons and primary afferent terminals in the spinal cord (25, 35), the preferential effect of JWH-133 on neuropathic pain is likely due to increased CB2 expression in DRG neurons and their central terminals at the spinal cord level.

A salient finding of our study is that activation of CB2 at the spinal cord level inhibits nociceptive glutamatergic input to dorsal horn neurons in neuropathic pain, which further supports the functional significance of nerve injury–induced CB2 upregulation in DRG neurons. Increased glutamatergic input to spinal dorsal horn neurons is critically involved in the maintenance of chronic neuropathic pain (19, 20, 23). CB2 agonists inhibit the hyperexcitability of spinal dorsal horn neurons in nerve-injured rats (36, 37). Our electrophysiological results performed in spinal cord slices showed that the CB2 agonist JWH-133 reduced the amplitude of EPSCs evoked from the dorsal root (*i.e.*, central terminals of DRG neurons) and increased the PPR of evoked EPSCs in spinal dorsal horn neurons of SNL rats. However, JWH-133 had no such effect in control rats. These data suggest that activation of presynaptic CB2 expressed at primary afferent central terminals inhibits glutamatergic input to spinal dorsal horn neurons augmented by nerve injury. Nerve injury can promote protein trafficking from DRG neurons to their central terminals in the spinal dorsal horn (19, 38, 39, 40). Consistent with our functional data using electrophysiological approaches, we showed that nerve injury significantly increased CB2 protein levels in the dorsal spinal cord synaptosomes. CB2 are coupled to Gαi proteins (41), and CB2 activation with CB65 and HU308 suppresses Ca^2+^ channel activity in retinal ganglion cells (42). Also, JWH-133 inhibits capsaicin-evoked calcium responses in DRG neurons (36). Thus, CB2 activation likely inhibits Ca^2+^ channels to reduce nociceptive input from primary afferents to spinal dorsal horn neurons in neuropathic pain.

Another new finding of our study is that a combination of coordinated histone modifications at the *Cnr2* promoter in the DRG is tightly correlated with CB2 upregulation induced by nerve injury. Histone modifications and DNA methylation are two major mechanisms involved in epigenetic control of gene transcription (29, 43, 44). In this study, we found that nerve injury increased the enrichment of two activating histone marks, H3K4me3 and H3K9ac, at the *Cnr2* promoter in the DRG. Strikingly, nerve injury also diminished the enrichment of two repressive histone marks, H3K9me2 and H3K27me3, at the *Cnr2* promoter in the DRG. However, our BSP and MeDIP-qPCR assays demonstrated that nerve injury did not alter the DNA methylation status at the *Cnr2* promoter in the DRG. These results are consistent with the genome-wide study showing a minimum effect of DNA methylation on nerve injury–induced expression of pronociceptive and antinociceptive genes in the DRG (17). H3K4me3 is present at most active promoters and is catalyzed by trithorax group proteins, whereas H3K27me3 is associated with silenced genes and induced by polycomb group proteins (45). Increased H3K4me3 enrichment at the *Cnr2* promoter in the DRG after nerve injury suggests a potential involvement of histone lysine methyltransferases. The catalytic addition of methyl groups to H3K4 residue in mammals is generally mediated by at least six Su(var)3-9, Enhancer of zeste, trithorax/mixed lineage leukemia (46). Furthermore, H3K9ac, regulated by histone acetyltransferases and deacetylases, is associated with transcriptional activation (29). On the other hand, H3K9me2, mediated by the G9a/G9a-like protein (GLP) histone methyltransferase, is usually involved in transcriptional repression (14, 15). Together, our findings suggest that coordinated differential changes in histone modifications at the *Cnr2* promoter are likely involved in nerve injury–induced CB2 upregulation in the DRG.

It is unclear how epigenetic regulators are organized to induce complex histone modifications at the *Cnr2* promoter in the injured DRG. H3K4me3 and H3K27me3 are the prominent histone marks of bivalency, controlled by both histone methyltransferase and demethylase activities (47, 48). Bivalent modifications by H3K4me3 and H3K27me3 were originally identified in embryonic stem cells (28) but have since been shown to play key roles in tissue- and cell type–specific gene expression associated with cellular phenotypical plasticity (49). The overlapping presence of both permissive H3K4me3 and repressive H3K27me3 histone marks at promoter regions keeps genes expressed at low levels but poised for rapid activation. Thus, although *Cnr2* with H3K27me3 enrichment is poorly transcribed in normal DRG, *Cnr2* transcription can be rapidly activated upon the loss of H3K27me3 by nerve injury, because of the remaining presence of H3K4me3. Intriguingly, we also found an increase in the enrichment of H3K9ac, paralleled by a reduction in H3K9me2, at the *Cnr2* promoter in the injured DRG, suggesting that the *Cnr2* promoter is also subjected to bivalent modifications by both H3K9ac and H3K9me2. Similar opposing changes of H3K9ac and H3K9me2 at the *Panx1* promoter have been shown in the injured DRG (16). Thus, the epigenetic bivalency of overlapping presence of both permissive and repressive histone modifications at gene promoters could be extended to H3K9ac and H3K9me2. The potential interplay between H3K9ac and H3K9me2 is not well defined, although inhibition of G9a/GLP activity increases global levels of H3K9ac while decreasing H3K9me2 in the hippocampus (50). RNA sequencing data showed that inhibition of G9a/GLP or histone deacetylases does not alter the CB2 expression level in the DRG (14, 51), suggesting that the activity of G9a/GLP and histone deacetylases alone may not play a major role in regulating CB2 expression in the DRG. Further studies are needed to identify complex interactions between various epigenetic regulators (*e.g.*, G9a/GLP, histone demethylases, trithorax group proteins, polycomb group proteins, and histone acetyltransferases and deacetylases) and transcriptional factors that lead to increased CB2 expression in the injured DRG.

In conclusion, our study provides new evidence about epigenetic regulation of CB2 expression in the DRG in neuropathic pain. The combined changes in histone modifications (*i.e.*, increased enrichment of H3K4me3 and H3K9ac and decreased levels of H3K9me2 and H3K27me3) at the *Cnr2* promoter are likely involved in nerve injury–induced CB2 expression in primary sensory neurons. Increased CB2 expression in DRG neurons and their central terminals underlies the inhibitory effect of the CB2 agonist on nociceptive transmission from primary afferents to spinal dorsal horn neurons in neuropathic pain. Thus, CB2 upregulation in DRG neurons offers a new analgesic target for treating neuropathic pain. CB2 agonists may be a better alternative to CB1 agonists, because CB2 agonists are nonpsychoactive and reduce neuropathic pain but not normal nociception.

## Experimental procedures

### Animal models

The experimental protocols and surgical procedures were approved by the Animal Care and Use Committee at the University of Texas MD Anderson Cancer Center. Male Sprague-Dawley rats (7–8 weeks old) were housed (2–3 rats/cage) on a standard 12:12 h light-dark cycle and received food and water *ad libitum*. SNL was performed as the experimental model of neuropathic pain as described previously (20, 52). Briefly, rats were anesthetized with 2 to 3% isoflurane, and then the left L5 and L6 spinal nerves were exposed and ligated separately with 6 to 0 silk sutures under a surgical microscope. In the SNL model, stable chronic pain typically develops 10 to 14 days after nerve injury and last more than 8 weeks (52, 53, 54). Sham surgery (the same surgical procedure without nerve ligation) was performed in rats and used as the control.

### Intrathecal drug administration

The CB2 agonist JWH-133 (#10005428, Cayman Chemical) was diluted in normal saline and injected intrathecally. The intrathecal injection was performed using a lumbar puncture technique, as previously described (35). Rats were briefly anesthetized with 2% isoflurane and then placed in a prone position with a tube under the abdomen to enlarge the lumbar vertebral space between L5 and L6. A lumbar puncture was performed with a 30-gauge needle connected to a 50-μl Hamilton syringe. Successful intrathecal injection (10 μl) of the drug solution was indicated by swift tail movement.

### Nociceptive behavioral tests

Mechanical allodynia was tested using von Frey filaments, as we described previously (55, 56). Rats were placed individually on a wire mesh platform and allowed to acclimatize for 30 min before the test. The filaments were applied perpendicular to the plantar surface with sufficient force to cause slight bending against the hindpaw for 6 s. Paw withdrawal, flinching, or paw licking was considered a positive response. When a response occurred, the filament of the next lower force was applied. In the absence of a response, the filament of the next greater force was applied. The tactile stimulus producing a 50% likelihood of withdrawal was determined using the ‘up-down’ method (52).

Mechanical nociception was measured using a paw pressure test, as we described previously (15, 55). In brief, a pressure stimulus was applied to the hindpaw by generating a constantly increasing force using a Randall–Selitto analgesiometer (Ugo Basile). When the animal displayed a withdrawal response, the device was immediately stopped. The nociceptive withdrawal threshold was recorded and read on the scale. A maximum of 400 g of pressure was used as a cutoff to avoid potential tissue injury.

Thermal hyperalgesia was measured by determining the withdrawal latency of the hindpaw in response to a radiant heat stimulus using a thermal testing apparatus (IITC Life Sciences). Rats were placed individually on an elevated glass plate and allowed to acclimatize for 30 min before the test. The temperature of the glass surface was maintained at 30 °C. A radiant heat stimulus was focused onto the plantar surface of the hindpaw through the glass plate. The latency was recorded when the rats lifted or licked the hindpaw. A cutoff time of 30 s was used to avoid tissue damage (15, 55). The investigators performing all the behavioral tests were blinded to the animal groups. All tests were done between 9 AM and 6 PM.

### Spinal cord slice preparation and recordings

Rats were anesthetized by inhalation of 2%–3% isoflurane. The lumbar spinal cord at the L4/L6 levels was obtained through laminectomy and then placed immediately in ice-cold sucrose artificial cerebrospinal fluid (aCSF) (234 mM sucrose, 3.6 mM KCl, 1.2 mM MgCl_2_, 2.5 mM CaCl_2_, 1.2 mM NaH_2_PO_4_, 12 mM glucose, and 25 mM NaHCO_3_, presaturated with 95% O_2_ and 5% CO_2_). Transverse slices (400 μm thick) were cut in ice-cold sucrose aCSF using a vibratome (Leica) and then transferred into Krebs solution (117 mM NaCl, 3.6 mM KCl, 1.2 mM MgCl_2_, 2.5 mM CaCl_2_, 1.2 mM NaH_2_PO_4_, 11 mM glucose, and 25 mM NaHCO_3_, gassed with 95% O_2_ and 5% CO_2_) at 34 °C for at least 1 h. For recordings, spinal cord slices were transferred into a glass-bottomed chamber on the stage of a microscope and perfused continuously with Krebs solution at 3 ml/min at 34 °C (57, 58).

Neurons from the lamina II outer region were identified under a microscope with a 60 × water-immersion objective and used for whole-cell voltage-clamp recordings (59, 60). A glass pipette electrode (4–7 MΩ) was filled with an internal solution containing 135 mM K-gluconate, 5 mM KCl, 2 mM MgCl_2_, 0.5 mM CaCl_2_, 5 mM Hepes, 5 mM EGTA, 5 mM Mg-ATP, 0.5 mM Na-GTP, and 10 mM lidocaine N-ethyl bromide (280–300 mOsm, pH 7.2–7.4). To measure glutamate release from primary afferent nerves, the dorsal root was electrically stimulated at 0.6 mA, 0.5 ms, and 0.1 Hz with a bipolar tungsten electrode placed at least 200 μm from the recorded neuron. Monosynaptic EPSCs were identified based on the constant latency and the absence of conduction failure of evoked EPSCs in response to a 20-Hz (lasting for 1 s) electrical stimulation (23, 61, 62). To determine PPR, two EPSCs were evoked by a pair of stimuli given at 50-ms intervals. The PPR was expressed as the ratio of the amplitude of the second synaptic response to the amplitude of the first synaptic response. EPSCs were recorded at a holding potential of -60 mV, and signals were recorded using an amplifier (MultiClamp700B, Axon Instruments Inc), filtered at 1 to 2 kHz, digitized at 10 kHz, and stored for off-line analysis. The input resistance was monitored, and the recording was abandoned when the input resistance changed by more than 15%. JWH-133 (10, 20, and 50 μM) was bath applied in an ascending order, each for 6 min. All drugs were freshly prepared in aCSF before the experiments and delivered *via* syringe pumps.

### Synaptosome preparation

Synaptosomes were isolated from the dorsal spinal cord at L5 and L6 levels from sham and SNL rats (21 days after surgery), as we described previously (19, 24, 40). Briefly, tissues were prepared using the Syn-PER reagent (#87793, Thermo Fisher Scientific) containing a Halt protease and phosphatase inhibitor cocktail (#78441, Thermo Fisher Scientific). The homogenate was centrifuged at 1200 g for 10 min at 4 °C to remove cell debris. The supernatant was centrifuged at 15,000 g for 20 min to obtain the crude synaptosome pellet, which was then resuspended in RIPA lysis and extraction buffer mixed with a Halt protease and phosphatase inhibitor cocktail for 30 min on ice. The lysate was cleared by centrifugation at 16,000*g* for 10 min at 4 °C to obtain the synaptosomal proteins for immunoblotting analysis using a rabbit anti-CB2 antibody (1:200) and a rabbit anti-PSD95 antibody (1:500; #3450S, Cell signaling Technology).

### Immunoblotting

The protein levels of CB2 in the dorsal spinal cord and DRG at L5 and L6 levels were quantified using immunoblotting. The tissues were harvested from rats deeply anesthetized with 5% isoflurane and flash-frozen in liquid nitrogen and stored at -80 °C. The protein was extracted using RIPA lysis and extraction buffer (#89900, Thermo Scientific) in the presence of a protease inhibitor cocktail (#A32955, Sigma-Aldrich). After brief pulses of sonication, the samples were incubated with lysis buffer on ice for 30 min and then centrifuged at 12,000*g* for 15 min at 4 °C. The supernatant was collected, and the protein concentration was measured using a DC Protein Assay Kit (#5000113, Bio-Rad). Thirty micrograms of protein were loaded and separated on 4 to 12% Mini Protein Gel (#A32955, Invitrogen) and transferred to a polyvinylidene difluoride membrane (#IPVH00010, MilliporeSigma). The membrane was incubated with blocking solution (5% nonfat dry milk in Tris-buffered saline and 0.05% Tween 20 (TBST)) at 22 °C for 1 h and then incubated overnight at 4 °C with an anti-rabbit CB2 antibody (1:200 dilution, #101550, Cayman Chemicals) diluted with TBST containing 3% bovine serum albumin. The specificity of the CB2 antibody for immunoblotting has been shown previously (8, 63). The membrane was washed three times using TBST and then incubated with horseradish peroxidase (HRP)-conjugated anti-rabbit IgG (1:10000; #7074S, Cell signaling Technology) for 1 h at 22 °C. The protein bands were detected using the SuperSignal West Pico Plus Chemiluminescent Substrate (#34580, Thermo Fisher Scientific). After stripping the bound antibodies with Restore Plus Western Blot Stripping Buffer (#46430, Thermo Fisher Scientific) at 25 °C for 10 min, this membrane was washed for 10 min with TBST and incubated with blocking solution at 25 °C for 1 h. The membrane was incubated with a rabbit anti-GAPDH antibody (1:7000, #5174S, Cell Signaling Technology) overnight at 4 °C followed by three 5-min washes, then incubated with HRP-coupled anti-rabbit IgG, and developed using chemiluminescence. The protein band intensity was visualized, quantified using Odyssey Fc Imager (LI-COR Biosciences), and normalized to GAPDH on the same gel.

### *RNAscope in situ* hybridization

We performed RNAscope *in situ* hybridization to detect CB2 mRNA and immunofluorescence labeling with NeuN (a neuronal marker) in L5 and L6 DRGs from sham and SNL rats. After being deeply anesthetized with 5% isoflurane, rats were transcardially perfused with sterile PBS and then 4% paraformaldehyde. DRGs were removed and postfixed in 4% paraformaldehyde overnight and then treated with 30% sucrose in PBS until the tissue settled at the base of the tube. Tissue was then embedded in M-1 Embedding Matrix (#1310, Thermo Fisher Scientific) and kept at −80 °C. These tissues were cut at 15 μm thick using a cryostat and mounted directly onto Superfrost Plus slides (#22-178-277, Fisher Scientific) and kept frozen until the RNAscope assay. Tissue slides were treated with the RNAscope Intro Pack for Multiplex Fluorescent Reagent Kit (#323137, Advanced Cell Diagnostics). The RNAscope target–specific oligonucleotide probe for CB2 (#315231) were designed by Advanced Cell Diagnostic. The HRP-C1 signal was developed using Opal-620 (#FP1495001 KT, Akoya Biosciences). The sections were then rinsed and incubated with the anti-NeuN antibody (#ab177487, 1:200, Abcam) diluted in PBS solution containing 3% bovine serum albumin and 0.3% Triton X-100 overnight at 4 °C. Subsequently, sections were rinsed in 0.1 M PBS and incubated with Alexa Fluor 488–conjugated donkey anti-rabbit IgG (#21206, 1:300, Invitrogen) for 1 h. The sections were then rinsed, mounted on slides, dried, and covered with coverslips. Images were acquired using a Zeiss confocal laser-scanning microscope at 40 × magnification. RNAscope signal was defined as punctate staining with little or no background (38). For quantitative imaging analysis using ImageJ, we randomly selected one image from each DRG and nine DRGs from each group. The CB2 mRNA punctate signals were first counted for each image and then compared between sham and SNL groups. We then classified DRG neurons into small (<30 μm), medium (30–50 μm), and large (>50 μm) groups based on their diameter and quantified the CB2 mRNA punctate signals per neuron (38).

### Quantitative PCR

The DRG tissues at L5 and L6 levels from the sham and SNL rats (21 days after surgery) were used to extract total RNA using the RNeasy Plus Universal Mini Kit (#73404, Qiagen). Equal amount of RNA was reverse transcribed followed by DNase digestion using the SuperScript IV VILO Master Mix with ezDNase Enzyme (#11766050, Invitrogen). For qPCR analysis, 10 ng of diluted cDNA was mixed with PowerUp SYBR Green Master Mix (#A25776, Applied Biosystems) in a 20 μl reaction volume (in triplicates). The fast cycling mode in a QuantStudio 7 Flex Real-Time PCR System (Applied Biosystems) with the following conditions were used: 50 °C for 2 min, 95 °C for 2 min, and 40 cycles of 95 °C for 1 s, and 60 °C for 30 s. The primers used for the *Cnr2* promoter variant identification are as follows: Exon 1 (forward), 5′-CAG GAC AAG GCT TCA CAA GA-3´; Exon 1 (reverse), 5′-ATG GAC AGA CAG GCT TTG G-3´; Exon 1´ (forward), 5′-GCC ACC CAG CAA ACA TCT A-3´; Exon 1´ (reverse), 5′-AGG CTT TGG CTG CTT CTA C-3´; Exon 2 (forward), 5′-AGC CAG CTC TAG GGT AAT G-3´; Exon 2 (reverse), 5′-ACA GAC TGC GTA AGG TAG G-3´; Gapdh (forward), 5′-AGA ATG GGA AGC TGG TCA TC-3´; Gapdh (reverse), 5′-CAG TAG ACT CCA CGA CAT ACT C-3´. The mRNA levels of the target variants were quantified using the comparative 2^−ΔΔCT^ method normalized to the Gapdh mRNA levels in the sham control samples.

### ChIP-qPCR assay

ChIP was performed using the Magna ChIP G Tissue kit (#17-20000, MilliporeSigma) with some modifications, as we described previously (14, 16). The frozen L5 and L6 DRGs (harvested 21 days after the sham or SNL surgery) were pooled from three rats into the stabilization buffer for 2 min at 22 °C. The tissues were then fixed using 1.5% formaldehyde (methanol-free) for 15 min at 22 °C and quenched using glycine. After three washes with ice-cold PBS containing a protease inhibitor cocktail, the tissue was homogenized in tissue lysis buffer and incubated for 15 min on ice. The nuclei were resuspended in ChIP Dilution buffer and sonicated (30 s ON, 45 s OFF, for 20 min at 85% amplitude) to obtain chromatin fragments of 200 to 800 bp using a water bath ultrasonicator (#Q700, QSonica) at 4 °C. The chromatin was cleared from insoluble materials by centrifugation and further diluted prior to overnight immunoprecipitation using the protein G magnetic beads and 5 μg of the following antibodies: rabbit anti-histone H3 (#2650S, Cell Signaling Technology), rabbit anti-H3K4me3 (#9751S, Cell Signaling Technology), rabbit anti-H3K9ac (#9649S, Cell signaling Technology), rabbit anti-H3K9me2 (#4658S, Cell Signaling Technology), and rabbit anti-H3K27me3 (#9733S, Cell Signaling Technology). The specificity of these antibodies has been determined previously (14, 15, 16, 64). One microgram rabbit IgG (#2729S, Cell Signaling Technology) was used as a negative control. An aliquot (10% of the volume used for ChIP per antibody) of the diluted chromatin was used as 10% input for normalization in the assay. The following day, chromatin (from both ChIP and input samples) was de-crosslinked, and DNA was purified using the QIAquick PCR purification kit (#58106, Qiagen). The DNA was used for qPCR using a QuantStudio 7 Flex System (Applied Biosystems) using the PowerUp SYBR Green Master Mix (#A25776, Applied Biosystems) and the following primers spanning the rat *Cnr2* promoter regions are as follows: *Cnr2* (-463/-373 bp) forward, 5′-CCA GGA ACT TGC CAC ATA GA-3´; *Cnr2* (-463/-373 bp) reverse, 5′-GCA CAC GCC TTT AAT CCT AAC-3´; *Cnr2* (-105/-15 bp) forward, 5′-TTC CAG AGG GCA TCT CTA TCT-3´; *Cnr2* (-105/-15 bp) reverse, 5′-GAT GGA CAT CCT AAC GAG AGA AC-3´; *Cnr2* (-53/+28 bp) forward, 5′-CTC TGT GCA TCC TGT TGT TCT C-3´; *Cnr2* (-53/+28 bp) reverse, 5′-TGT TCC TGT CCA GAG TGA GTA G-3´; *Cnr2* (+117/+219 bp) forward, 5′-GGG TAA GGC ATT CCC TAA CAG-3´; *Cnr2* (+117/+219 bp) reverse, 5′-AGG CCA GTT TAG GCA ACA TAG-3´. The Rat Negative Control Primer Set 1 (#71024, Active Motif) amplifying a 92 bp region from a gene desert on rat chromosome 3 was used as a control for the ChIP assay. The following PCR conditions were used as follows: 50 °C for 2 min, 95 °C for 2 min, and 40 cycles at 95 °C for 15 s, and 60 °C for 1 min. The threshold cycle (C_T_) value in each group was normalized to the input using the following formula: (2^-ΔC^_T_) x 100%; where ΔC_T_ represents (C_T_ [ChIP] – (C_T_ [Input] - Log2 (Input Dilution Factor). The data were normalized using total histone H3 values.

### DNA methylation analysis

The methylation status of the CpG dinucleotides present at the *Cnr2* promoter was determined using BSP and MeDIP-qPCR. The methylated CpGs of the L5 and L6 DRG tissues, collected from rats 21 days after sham or SNL surgery, were compared. The BSP was performed as we reported previously (14, 16) with the following modifications. Briefly, genomic DNA from DRG tissues was extracted and immediately treated with bisulfite solution using the EpiTect Fast LyseAll Bisulfite Kit (#59864, Qiagen). This treatment converts most of the cytosines into uracil except the methylated cytosines of CpG dinucleotides in the presence of the DNA protect buffer that prevents degradation of genomic DNA. Using specific primers, the -300 bp to -108 bp and -66 bp to +134 bp region of the CB2 promoter were amplified using EpiMark Hot Start Taq DNA Polymerase (#M0490S, New England Biolabs). The following primers were used for the qPCR: *Cnr2* (-300/-108 bp) forward, 5′-AGA AAT TAT AAT GAG TGT TTT TGG TTT A-3’; *Cnr2* (-300/-108 bp) reverse, 5′-CAC ATA AAT ACC TTC TAA AAA ACC TAT T-3’; *Cnr2* (-66/+134 bp) forward, 5′-GGG TTT TTT GTT GTT TTG TGT ATT T-3’; *Cnr2* (-66/+134 bp) reverse, 5′-TTA AAA AAT ACC TTA CCC CAT TCA A-3’. The amplified PCR product was then cloned into the Chemically Competent *Escherichia coli* (One Shot TOP10) cells using the TOPO TA Cloning Kit for Sequencing (#K457501, Invitrogen). The plasmid DNA was isolated from at least six individual colonies of bacterial clones for both regions and sequenced. Each sequence representing different alleles was analyzed and mapped to the corresponding PCR amplified genomic DNA region for the presence of methylated CpGs using the quantification tool for methylation analysis (65).

For MeDIP-qPCR, we used the MeDIP Kit (#55009; Active Motif), and each sample contained pooled L5-L6 DRG tissues from two rats subjected to sham or SNL. First, the genomic DNA from the DRGs (after 21 days of sham/SNL) was isolated using the DNeasy Blood & Tissue Kit (#69504, Qiagen) according to the manufacturer’s instructions, except that the isolated genomic DNA was also subjected to RNase (DNase-free) treatment for residual RNA removal. Twenty micrograms of purified genomic DNA (diluted to 300 μl with 10 mM Tris–HCl, pH 8.5) was immediately sonicated with eight pulses of 15 s ON and 45 s OFF at 40% amplitude on ice to produce fragments of 200 to 600 bp, confirmed on a 1% agarose gel. A 50 ng of the fragmented DNA was set aside as 10% input DNA for normalization. About 500 ng of the fragmented DNA (in duplicates) was denatured at 95 °C for 10 min and immediately cooled on ice. The fragmented and denatured DNA was immunoprecipitated overnight using 2 μg of either mouse IgG (the negative control) or mouse anti–5-mC antibody in the presence of the bridging antibody (2 μg) and protease inhibitor cocktail provided in the kit. This 5-mC monoclonal antibody allows precipitation of only 5-mC–containing ssDNA (31). Using protein G–magnetic beads, the captured chromatin was eluted, treated with 1 μg Proteinase K, and finally purified using the QIAquick PCR Purification Kit (#28104, Qiagen). The purified DNA was used for MeDIP-qPCR using the following primers: *Cnr2* (-263/-128 bp) forward, 5′-CCC TAA GAA TCA GTG GGC ATT-3’; *Cnr2* (-263/-128 bp) reverse, 5′-GAC CTA TTT CTA GGG CAA GG-3’; *Cnr2* (-106/+21 bp) forward, 5′-CTT CCA GAG GGC ATC TCT ATC-3’; *Cnr2* (-106/+21 bp) reverse, 5′-GTC CAG AGT GAG TAG GAA AGG-3’. The following PCR conditions were used as follows: 50 °C for 2 min, 95 °C for 2 min, and 40 cycles of 95 °C for 15 s, and 60 °C for 1 min; a melt curve analysis was used for confirming amplicon specificity of the primers. Using the C_T_ values of a serially diluted purified input DNA (10%), a standard curve was prepared to obtain the concentration of enriched DNA. The percent of enrichment of 5-mC–containing DNA in each genomic region in the immunoprecipitated DNA by the 5-mC antibody or IgG was calculated from the input and plotted for each region.

### Statistical analysis

All data are expressed as means ± SEM. Evoked EPSCs were analyzed using Clampfit 10.0 software (Molecular Devices), and the amplitude of evoked EPSCs was quantified by averaging six consecutive EPSCs. One neuron from each spinal cord slice was recorded, and 4 to 5 rats were used for each group. A two-tailed Student’s *t* test was used to compare two groups. Mann-Whitney U test was used to compare two groups for the MeDIP–qPCR results. To determine the differences between more than two groups, one-way or two-way ANOVA followed by Tukey’s or Dunnett’s *post hoc* test was used. Behavioral data were compared using repeated measures ANOVA followed by Dunnett’s *post hoc* test. All statistical analyses were performed using Prism software (version 8, GraphPad Software Inc). *p* < 0.05 was considered to be statistically significant.

## Data availability

Raw data will be made available upon reasonable request.

## Conflict of interest

The authors declare no competing financial interest.
